# Local Bone Mineral Density, Subcutaneous and Visceral Adipose Tissue Measurements in Routine Multi Detector Computed Tomography—Which Parameter Predicts Incident Vertebral Fractures Best?

**DOI:** 10.3390/diagnostics11020240

**Published:** 2021-02-04

**Authors:** Egon Burian, Lioba Grundl, Tobias Greve, Daniela Junker, Nico Sollmann, Maximilian Löffler, Marcus R. Makowski, Claus Zimmer, Jan S. Kirschke, Thomas Baum

**Affiliations:** 1 Department of Diagnostic and Interventional Neuroradiology, Klinikum Rechts der Isar, TUM School of Medicine, Technical University of Munich, Ismaninger Str. 22, 81675 Munich, Germany; lioba.grundl@tum.de (L.G.); tobias.greve@med.uni-muenchen.de (T.G.); nico.sollmann@tum.de (N.S.); m.loeffler@tum.de (M.L.); claus.zimmer@tum.de (C.Z.); jan.kirschke@tum.de (J.S.K.); thomas.baum@tum.de (T.B.); 2 Department of Diagnostic and Interventional Radiology, Klinikum Rechts der Isar, Technical University of Munich, Ismaninger Str. 22, 81675 Munich, Germany; daniela.junker@tum.de (D.J.); marcus.makowski@tum.de (M.R.M.); 3 Department of Neurosurgery, Ludwig-Maximilians-University, Marchioninistraße 15, 81377 Munich, Germany

**Keywords:** incident vertebral fractures, CT, BMD, osteoporosis, SAT, VAT

## Abstract

In this case-control study the value of bone mineral density (BMD) at different vertebral levels, subcutaneous adipose tissue (SAT), and visceral adipose tissue (VAT) to identify patients with incident osteoporotic vertebral fractures in routine multi-detector computed tomography (MDCT) exams was assessed. Material and methods: Seventeen patients who underwent baseline and follow-up routine contrast-enhanced MDCT and had an incident osteoporotic vertebral fracture at follow-up were included. Seventeen age-, sex- and follow-up duration-matched controls were identified. Trabecular BMD (from Th5 to L5) as well as cross-sectional area of SAT and VAT were extracted. Results: BMD performed best to differentiate patients with an incident fracture from controls at the levels of Th5 (area under the curve [AUC] = 0.781, *p* = 0.014), Th7 (AUC = 0.877, *p* = 0.001), and Th9 (AUC = 0.818, *p* = 0.005). Applying multivariate logistic regression BMD at Th7 level remained the only significant predictor of incident vertebral fractures (Th5-L5) with an odds ratio of 1.07 per BMD SD decrease. VAT and SAT did not show significant differences between the fracture and control group (*p* > 0.05). Conclusion: The local BMD measurement appears to be more suitable than standard mean BMD from L1–L3 for fracture risk assessment.

## 1. Introduction

### 1.1. Background

Osteoporosis is broadly known as a systemic metabolic bone disease, which is accompanied by bone mass decrease and rarefication of the trabecular structure [1]. This disease frequently manifests itself in form of fragility fractures, including the hip and spine as the major sites of fracture occurrence. These vertebral and femoral fractures increase the individuals’ morbidity and mortality significantly and drastically impair patients’ quality of life [2,3,4]. 43.4 million older adults suffered from loss of bone mass in the osteopenic to osteoporotic range in the US [5]. In Europe total fragility fractures are estimated to increase from 2.7 million in 2017 to 3.3 million in 2030; a 23%. The resulting annual fracture-related costs (€37.5 billion in 2017) are expected to increase by 27% [6].

A precise and valid diagnostic algorithm should be established identifying subjects at risk for an osteoporotic fracture, to enable the clinician to initiate medication adjustments at an early stage [4,7,8]. In order to address the question of which individual is prone to fractures the Fracture Risk Assessment Tool (FRAX^®^, University of Sheffield, Sheffield, UK) has been generated [7,9,10]. Based on clinical risk factors and areal bone mineral density (aBMD) at the femoral neck, this score allows for 10-year fracture risk assessment [9]. Dual-energy X-ray absorptiometry (DXA) is the reference technology to quantify areal BMD in grams per cm^2^ [11]. Quantitative computed tomography (QCT) can evaluate volumetric BMD in cortical and trabecular bone, separately, and can provide improved fracture risk assessment compared to DXA [12,13]. Moreover, routinely performed multi-detector computed tomography (MDCT) for purposes other than osteodensitometry, e.g., cancer staging, allow for opportunistic osteoporosis screening by assessing BMD without additional costs or radiation exposure [11,14]. However, previous studies investigated only mean lumbar BMD in routine MDCT to predict incident vertebral fractures [12,15].

In the past, multiple studies investigated the impact of further imaging-based parameters like major fat depots and large muscle groups of the human body on fracture risk beyond BMD. As a consequence there is plentitude of literature on the subcutaneous adipose tissue (SAT) and visceral adipose tissue (VAT), as well as paraspinal muscle characteristics on fracture risk assessment [16,17,18,19]. However, the reported results on the relationship between the different fat depots and BMD were contradictory in great parts [20,21]. Recently, Beaudoin et al. reported the highest discriminative power for BMD regarding predicting fractures, but the current data on other factors including body fat distribution patterns like VAT/SAT influencing the probability for incident fractures is still unclear [22,23]. In a lately conducted meta-analysis on the influence of body mass index (BMI) on vertebral fracture overall risk, Kaze et al. reported a certain heterogeneity among the analyzed studies, suggesting to further explore body fat distribution patterns and their association with vertebral fractures [23].

### 1.2. Objectives

The goal of this case-control study was to evaluate the performance of BMD at different vertebral levels, SAT, and VAT acquired with routine MDCT for future fracture risk assessment in an oncologic cohort. We hypothesized that local BMD measurements in combination with SAT and VAT assessment improve the prediction of osteoporotic vertebral fractures beyond the gold standard of mean L1–3 BMD measurements.

## 2. Materials and Methods

### 2.1. Participants and Setting

Patients with a baseline and at least 6 months follow-up routine contrast-enhanced MDCT (Somatom Sensation Cardiac 64; Siemens Medical Solutions, Erlangen, Germany) of the thorax and abdomen were extracted from the institutional picture archiving and communication system (PACS, Sectra AB; Linköping, Sweden) retrospectively. All patients suffered from an incident osteoporotic vertebral fracture at follow-up. Baseline imaging had to be performed at one specific MDCT scanner with the same protocol as outlined below. The patients were in chemotherapeutic treatment due to oncologic underlying diseases (such as esophageal, colorectal, or breast cancer). Six subjects from the fracture group suffered from esophageal cancer, 4 subjects had gastric cancer, 2 had colorectal cancer, 2 patients had duodenal cancer, 2 had breast cancer, and one patient suffered from Hodgkin lymphoma. In the control group the underlying oncologic diseases were as follows: 10 patients had esophageal cancer, 4 subjects had gastric cancer, 2 had colorectal, and one patient had duodenal cancer. All patients included in this study were recruited within the oncological follow-up examination which includes diagnostic MDCT scans to exclude tumor recurrence. In course of the treatment of the underlying oncologic disease all included patients received chemotherapy. No history of bisphosphonates or bone turnover influencing medication was known due to medical reports. In course of dedicated oncologic long term follow-up examination the patients received periodic MDCT scans to detect potential tumor recurrence. For this study, all patients with osseous metastasis, but also hematological or metabolic bone disorders aside from osteoporosis were excluded. All acquired medical data and reports were scanned for existing osseous alterations indicating tumorous infiltration.

### 2.2. Study Design

Seventeen patients with incident vertebral fractures were included in the present study. Furthermore, 17 age-, sex-, and follow-up duration-matched patients with no baseline and follow-up vertebral fractures were identified as controls.

Before study initiation approval of the local institutional review board was obtained (27/19S). Overall, all research activities reported in this work were performed according to the Declaration of Helsinki and according to Good Clinical Practice. All patients included in this case-control study gave written informed consent for analytic workup of the individual data before each MDCT scan.

### 2.3. Multi Detector Computed Tomography (MDCT) Imaging

All MDCT scans were performed using a 64-row MDCT scanner (Somatom Sensation Cardiac 64; Siemens Medical Solutions, Erlangen, Germany). Standardized protocols with dedicated scanning parameters were applied. An average 120 kVp tube voltage with an adapted tube load of averaged 200 mAs and minimum collimation (0.6 mm) were delivered. For enhancing soft tissue contrast intravenous contrast medium (Imeron 400; Bracco, Konstanz, Germany) was administered in each examination following a standardized protocol using a high-pressure injector (Fresenius Pilot C; Fresenius Kabi, Bad Homburg, Germany) with a delay of 70 s, a flow rate of 3 mL/s, and a body weight-dependent dose (80 mL for body weight up to 80 kg, 90 mL for body weight up to 100 kg, and 100 mL for body weight over 100 kg). Before each MDCT scan all patients were given oral contrast medium (1000 mL of Barilux Scan; Sanochemia Diagnostics, Neuss, Germany) for better discrimination of the intestinal organs. To enable subsequent BMD calculation a density reference phantom (Osteo Phantom; Siemens Medical Solutions) was integrated in the scanning procedure.

Prevalent and incident vertebral fractures were diagnosed in the sagittal reformations of the MDCT scans by a radiologist with nine years of experience.

The reference phantom was placed continuously in the table mat of scanner to perform a synchronous calibration for opportunistic BMD assessment like described before by Bauer et al., and Baum et al. [24,25].

### 2.4. Quantitative Variables

#### 2.4.1. Bone Mineral Density (BMD) Measurements

**Figure 1:**
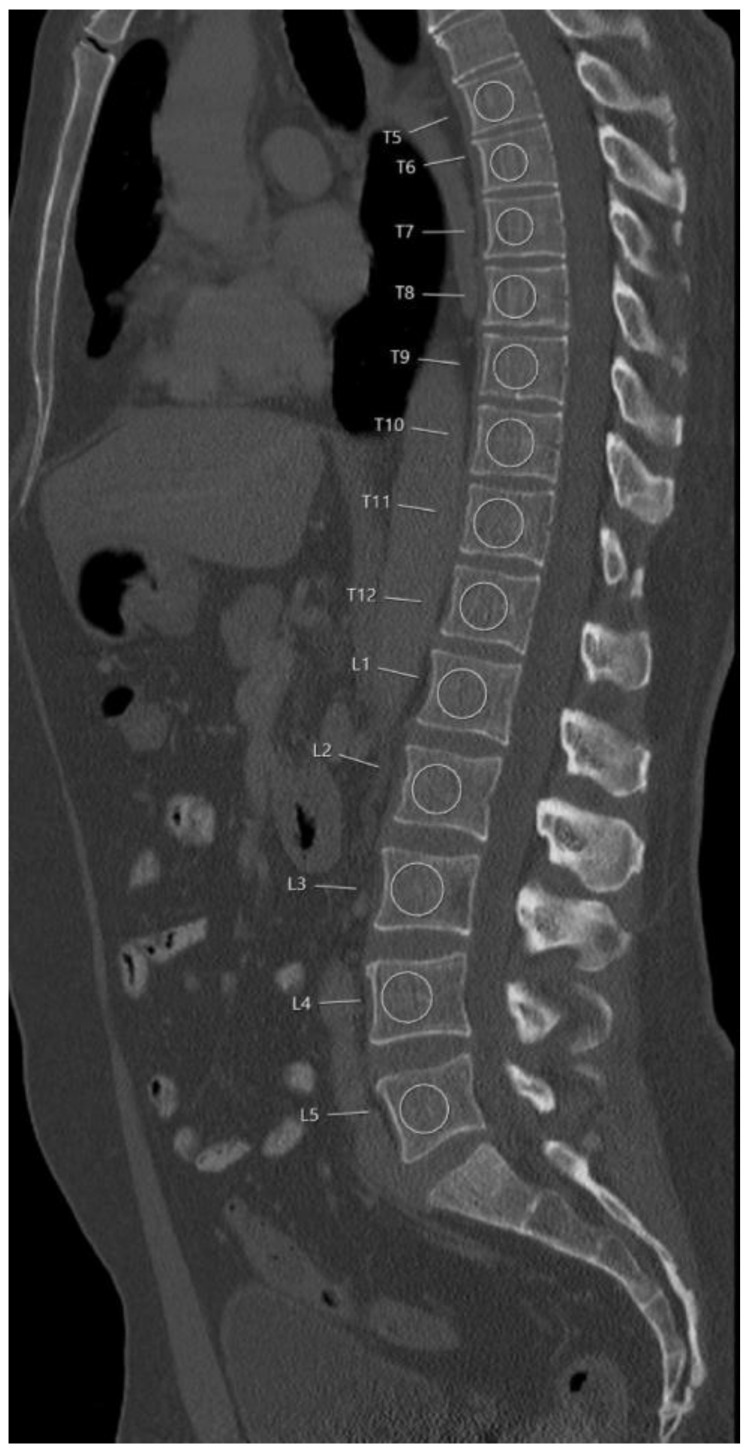
Representative placement of the regions of interest (ROIs). ROIs were placed in the ventral halves of the trabecular compartment of the vertebral bodies, equidistant to both endplates, excluding the venous plexus.

For BMD measurements in the baseline MDCT, circular regions of interest (ROIs) were defined and placed in vertebral bodies from Th5 to L5. Using the institutional PACS (Sectra AB; Linköping, Sweden) sagittal reformations of the spine with a slice thickness of 3 mm were selected for this purpose. In the most central slice depicting the vertebral body, ROIs were placed in the trabecular compartment of the anterior vertebral body excluding the cortical structures of the endplates. Additionally, inclusion of the vertebral venous plexus on BMD measurements was avoided. Each ROI diameter was standardized to two-thirds of the vertebral height (Figure 1). This approach has been reported previously [15,25]. BMD was not measured in vertebrae with prevalent fracture.

The extracted Hounsfield Units (HU) were converted to volumetric BMD (in mg/cm^3^) using synchronous calibration. For this conversion, HUs in the two phases of the reference phantom in the scanner couch were manually sampled in ROIs placed by one radiologist with two years of experience. The placement of the ROIs and BMD calculation took approximately 3 min for each patient. Following this approach, a linear HU-to-BMD-conversion equation was established for each patient scan. For this measurement technique Baum et al. previously reported reproducibility errors of 2.09% to 7.70% [15,25].

#### 2.4.2. Subcutaneous Adipose Tissue (SAT) and Visceral Adipose Tissue (VAT) Volume Quantification

**Figure 2:**
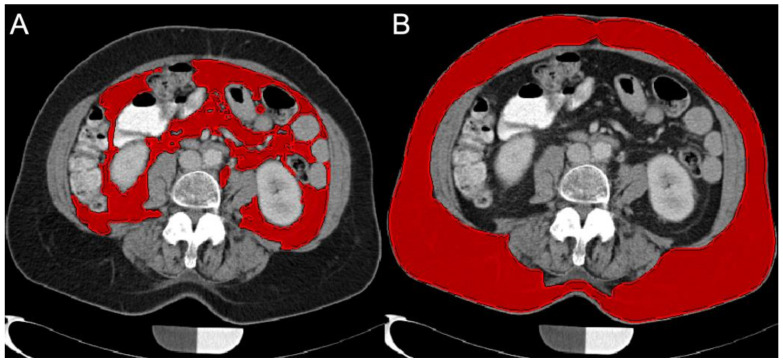
Representative segmentation for visceral adipose tissue (VAT; (**A**)) and subcutaneous adipose tissue (SAT; (**B**)) is shown at the level of L4/5.

For SAT and VAT calculations, axial reformations with 5 mm slice thickness were used. Images were loaded in MITK (Medical Imaging Interaction Toolkit; mitk.org). The slice at the level of L4/5 was identified and two adjacent slices cranial and caudal, respectively, were included in the fat volume quantification. These regions were repeatedly used for SAT/VAT quantification in literature and pose standardized localizations for quantitative volumetric assessment [19,26]. SAT-VAT segmentation was performed manually assisted by a region growing based algorithm, operating threshold and pattern based. SAT volume defined as the volume circumscribed by the abdominal cutis as the outer border and the abdominal and paravertebral musculature as the inner border. The VAT volume was identified and quantified according to Sheu et al. [18]. The borders of the local musculature were identified and excluded from the SAT/VAT segmentation. The VAT was defined as the sum of all voxels within the inner border, which were neither muscle nor intestinal tissue and were in the adipose tissue range. SAT was defined as the voxels within the outer and inner border, the tissue from the cutis to the muscle tissue was included (Figure 2). All image analysis steps were performed by a radiologist with three years of experience.

### 2.5. Statistical Methods

Statistical interpretation and data analysis were performed using SPSS (version 25; SPSS Inc., Chicago, IL, USA). All tests were done using a two-sided 0.05 level of significance. Prior to statistical work up distribution testing of the acquired results was performed. Applying the Shapiro–Wilk-test non-Gaussian distribution for the majority of parameters was revealed. Means and standard deviation (SD) of BMD at vertebral levels from Th5 to L5, BMD averaged over L1–L3 (as the QCT standard), SAT, VAT, and VAT/SAT ratio were calculated and compared between patients with incident fractures and controls using the Wilcoxon signed-rank test.

Correlations of BMD, SAT, VAT, and VAT/SAT ratio were analyzed using Spearman’s correlation coefficients. Odds ratios with 95% Confidence Interval (CI) as well as receiver operating characteristics (ROC) analyses were performed and the area under the curve (AUC) and its standard error (SE) were calculated to evaluate the diagnostic performance of BMD, SAT, VAT, and VAT/SAT ratio to differentiate patients with incident fractures from controls. The analyses have been done similar to Muehlematter et al. and Valentinitsch et al. [27,28]. Furthermore, multivariate logistic regression models were used to determine significant predictors of incident fractures. Parameters (baseline fracture status, BMD, SAT, VAT, and VAT/SAT ratio) were included in a stepwise approach in the regression models if the level of significance was *p* < 0.05. The performance of a significant predictor was expressed as odds ratio and 95% confidence interval per SD decrease of the respective predictor.

## 3. Results

### 3.1. Participants

Seventeen patients (8 men, 9 women, age: 65.8 ± 9.2 years) who underwent baseline and follow-up routine thoracic and abdominal contrast-enhanced MDCT and had an incident osteoporotic vertebral fracture at follow-up were included.

Five of 17 included patients had a prevalent vertebral fracture at baseline at the levels of Th7, Th10, Th12, L2, and L4, respectively. Six of the included patients showed an incident fracture at the level of Th12, two patients each had a fracture at Th7, Th11, L1, and L3. Respectively, one patient each showed a fracture at the level of Th9, L4, and L5. Due to the matching, no significant (*p* > 0.05) differences were observed for age and follow-up time between the fracture group (65.9 ± 9.4 years and 20.1 ± 10.6 months follow-up) and the control group (66.7 ± 9.0 years and 22.7 ± 12.5 months follow-up).

### 3.2. Descriptive Data

Vertebral body BMD at the levels Th7 (*p* = 0.008), Th9 (*p* = 0.005), Th11 (*p* = 0.041), and Th12 (*p* = 0.041) showed significant differences between the two groups. Neither SAT, VAT nor the VAT/SAT ratio showed significant differences between the groups (Table 1).

### 3.3. Outcome Data

All vertebral bodies from Th5 to L5 showed significant correlations among each other (ranging from r = 0.445, *p* = 0.011 for BMD of Th7/L2, to r = 0.855, *p* < 0.001 for BMD of Th5/Th11). The mean BMD of L1-L3 also significantly correlated with all other vertebral levels (r > 0.66, *p* < 0.001). Significant correlations between VAT and BMD were only found at the levels of L1 (r = 0.401, *p* = 0.021), L2 (r = 0.445, *p* = 0.011), and for the mean BMD of L1-L3 (r = 0.403, *p* = 0.022). Neither SAT nor VAT/SAT ratio revealed significant correlations with BMD at any vertebral level (*p* > 0.05).

### 3.4. Main Results

BMD at all levels except of Th6 revealed significant AUCs, with Th5 (AUC = 0.781, *p* = 0.014), Th7 (AUC = 0.877, *p* = 0.001), and Th9 (AUC = 0.818, *p* = 0.005) showing the highest AUC values. BMD from all lumbar levels as well as measurements for SAT, VAT, and VAT/SAT ratio did not display an AUC on a significant level (*p* > 0.05) (Table 2).

In multivariate logistic regression models, BMD at Th7 level remained the only significant predictor of incident vertebral fractures for all included vertebral levels (Th5-L5), with an odds ratio of 1.07 per BMD SD decrease (95% confidence interval: 1.01–1.14).

## 4. Discussion

In this case-control study, we assessed the ability of local BMD, VAT, and SAT measured in routine MDCT to predict incident vertebral fractures. BMD extracted from the vertebral body Th7 and not the mean BMD from L1 to L3 showed the highest discriminative power to differentiate individuals suffering from an incident fracture from controls. VAT and SAT yielded no significant information on prospective vertebral fracture occurrence.

The reference technique for bone densitometry used in the diagnosis of osteoporosis and the assessment of fracture risk remains DXA since its endorsement by the World Health Organization (WHO) more than 25 years ago [29]. However, QCT has evolved as an alternative, non-projectional imaging technique performed on clinical CT scanners [13]. Over the last 20 years, a large amount of studies has been published on opportunistic BMD screening using routine MDCT scans that have been acquired for purposes other than bone densitometry [11,15,24,25]. In case of contrast-enhanced MDCT scans, e.g., for cancer staging and follow-up, conversion equations and correction offsets have been described [15,25].

The prospect of identifying subjects with certain regionally distinct BMD distribution patterns for being at risk for vertebral fracture occurrence offers an attractive, non-invasive possibility of using MDCT beyond mere BMD calculation. Recently, Allaire et al. compared different parameters circumscribing osseous mineral content (integral BMD, trabecular BMD, and vertebral compressive strength, amongst others) at the level of L3 using finite element analysis with regard to their potential to predict incident vertebral fractures [30]. They reported an AUC with higher discriminative power for the level L3 compared to this study (Allaire et al.: AUC (L3) = 0.815; present study: AUC (L3) = 0.663), but revealed a lower AUC compared to the vertebral body we identified as the most reliable predictor for prospective fractures from Th5-L5 (AUC (Th7) = 0.877) [30]. Our presented study is the first to expand the vertebral levels of BMD measurements from Th5 to L5 in an oncologic population. The results highlight the importance of regional BMD assessment for clinical decision-making about antiresorptive treatment for the purpose of primary and secondary fracture prevention in cancer patients [31].

The stability of the vertebral column is not only dependent on the mineral content of the bone but also on the soft tissue composition. Although the crucial importance of BMD for fracture risk assessment was investigated in the past by various groups with rather homogenous results, the significance of morphological parameters of body composition is not completely clarified [10,17,22,23,32,33]. The occurrence of catabolic conditions and low body weight are risk factors for decreasing BMD and increasing fracture incidence [34,35]. Despite the pathophysiological knowledge of obesity leading to increased central adiposity and low-grade systemic inflammatory processes which, in consequence, have a negative impact on bone metabolism, the impact of VAT and SAT on vertebral fracture risk is still not understood [20,35]. The complexity of the interaction between hormonal regulatory mechanisms accompanying fat accumulations is elucidated by de Araujo et al. and de Frauda et al., who described the negative association of insulin resistance and VAT on bone quality and BMD [21,36]. Taken together, the recent literature emphasizes the non-linear interaction paths and feedback loops of energy metabolism and bone mineral homeostasis [21]. Although some groups reported an inverse relationship between VAT or VAT/SAT ratio with BMD values, the existing studies were limited to healthy, predominantly female subjects [20,21,37]. Thus, our results indicating no significant correlations between VAT or SAT and BMD in an oncologic cohort with a balanced male-to-female ratio can only be compared to these studies with caution.

The strength of our study lies in the level-dependent analysis of BMD distribution with regard to prediction of prospective incidental fractures, which has not been investigated before and has the possibility for implementation in clinical routine. Although, there have been approaches to identify patients at the risk for vertebral fractures with texture analysis derived from MDCT scans, so far the impact of regional BMD heterogeneities in this context remains an issue of high scientific and clinical interest [27,28]. The presented results show the potential of regional BMD measurements and highlight the clinical applicability in routine MDCT scans with immediate implications for patient care. Supporting the trend our results indicate, Pickhardt et al. have shown recently, that regional fat distribution patterns play a rather subordinate role in fracture risk assessment compared to local BMD [38].

This study has some limitations. First, the relatively small sample size is a major limitation of the study. Second, underlying oncologic disease entities and treatment regimen are potential confounding factors affecting BMD and osseous changes in microstructure due to medication and cancer-related causes which were not adjusted for in the present study. Third, beyond SAT and VAT this study did not include further body measures like BMI as they were not collected on a routine basis. Future investigations may refine the results of this study by including other parameters beyond SAT, VAT, and VAT/SAT ratio. As shown by Nielson and Paik before, obesity and BMI on constant BMD can predispose individuals to future fracture occurrence and therefore pose important risk factors in a multiparametric fracture risk assessment approach [17,32]. Fourth, given the oncologic cohort there is a certain probability of bone marrow micro metastases, which cannot be detected in routine MDCT but have an impact on bone homeostasis and turnover [39]. In consequence the biomechanical stability might be impaired to a not visualizable extent. Last, vitamin D status was not monitored in the subjects included in this study.

Last, assessment of BMD (change) using routine MDCT data is often not possible with an acceptable reproducibility error. However, we used the same scanner with an identical protocol and performed synchronous calibration with a reference phantom in all scans. Thus, a robust BMD-based prediction of osteoporotic fracture risk should be ensured.

## 5. Conclusions

The presented study showed that BMD at the vertebral level of Th7 revealed the best discriminative power to identify patients with incident vertebral fractures. This local BMD measurement appears to be more suitable than standard mean BMD from L1–L3 for fracture risk assessment and can be implemented easily in clinical routine MDCT examinations. SAT and VAT were no significant risk factors for incident vertebral fractures.

## Figures and Tables

**Table 1:** Bone mineral density (BMD) is displayed from Th5 to L5. Subcutaneous adipose tissue (SAT) and visceral adipose tissue (VAT) are measured at the level L4/5. Parameters were compared between the two groups with Wilcoxon-signed rank-tests.

Parameters	Status	Mean	SD	*p*
Age (years)	0	66.7	9.0	0.972
	1	65.9	9.4	
Follow-up (months)	0	22.7	12.5	0.543
	1	20.1	10.6	
Th5 (mg/mL)	0	176.4	33.1	0.079
	1	142.2	17.9	
Th6 (mg/mL)	0	165.3	39.7	0.234
	1	132.8	32.5	
Th7 (mg/mL)	0	166.3	22.3	0.008
	1	128.4	32.5	
Th8 (mg/mL)	0	151.4	30.4	0.061
	1	123.4	27.6	
Th9 (mg/mL)	0	158.9	20.9	0.005
	1	124.1	27.5	
Th10 (mg/mL)	0	162.2	29.7	0.112
	1	135.7	27.4	
Th11 (mg/mL)	0	154.0	25.5	0.034
	1	129.4	20.5	
Th12 (mg/mL)	0	141.7	26.9	0.041
	1	116.3	16.0	
L1 (mg/mL)	0	136.8	32.4	0.121
	1	115.1	25.1	
L2 (mg/mL)	0	137.4	32.6	0.088
	1	113.3	21.7	
L3 (mg/mL)	0	127.1	30.3	0.278
	1	111.9	19.7	
L4 (mg/mL)	0	128.8	31.7	0.569
	1	113.6	25.6	
L5 (mg/mL)	0	133.3	47.9	0.955
	1	117.9	40.1	
L1-L3 (mg/mL)	0	133.8	29.4	0.140
	1	112.6	19.5	
VAT (cm^3^)	0	266.3	262.7	0.589
	1	245.0	260.2	
SAT (cm^3^)	0	519.3	339.8	0.829
	1	514.0	347.5	
VAT/SAT	0	0.6	0.6	0.914
	1	0.5	0.5	

Abbreviations: 0 = control group; 1 = fracture group; L: lumbar vertebral body; SAT: subcutaneous adipose tissue; SD = standard deviation. Th: thoracic vertebral body; VAT: visceral adipose tissue.

**Table 2:** The odds ratios with 95% Confidence Interval per BMD SD decrease as well as area under the curve values of the areal bone mineral density from Th5 to L5, subcutaneous adipose tissue and visceral adipose tissue in patients with incident fractures versus controls.

Parameters	Odds Ratio	CI	AUC	SE	*p*
Th5	1.05	1.01–1.09	0.781	0.090	0.014
Th6	1.03	1.00–1.05	0.722	0.097	0.051
Th7	1.07	1.01–1.14	0.877	0.066	0.001
Th8	1.04	1.01–1.07	0.738	0.099	0.036
Th9	1.06	1.02–1.10	0.818	0.089	0.005
Th10	1.03	1.01–1.06	0.754	0.100	0.025
Th11	1.04	1.01–1.08	0.754	0.094	0.025
Th12	1.05	1.01–1.10	0.749	0.096	0.029
L1	1.03	1.00–1.05	0.668	0.106	0.138
L2	1.03	1.01–1.06	0.658	0.104	0.165
L3	n.s.		0.663	0.104	0.151
L4	n.s.		0.604	0.111	0.359
L5	n.s.		0.481	0.115	0.869
L1-L3	1.04	1.01–1.07	0.684	0.104	0.105
VAT	n.s.		0.578	0.112	0.495
SAT	n.s.		0.497	0.116	0.981
VAT/SAT	n.s.		0.519	0.118	0.869

Abbreviations: AUC: area under the curve; CI: confidence interval; L: lumbar vertebral body; n.s.: not significant; SAT: subcutaneous adipose tissue; SD = standard deviation; SE = standard error, Th: thoracic vertebral body; VAT: visceral adipose tissue.

## Data Availability

The data presented in this study are available on request from the corresponding author. The data are not publicly available due to patient privacy.
